# Monounsaturated fatty acid, carbohydrate intake, and diabetes status are associated with arterial pulse pressure

**DOI:** 10.1186/1475-2891-10-126

**Published:** 2011-11-16

**Authors:** Joan A Vaccaro, Fatma G Huffman

**Affiliations:** 1 Florida International University, Robert Stempel College of Public Health and Social Work, Department of Dietetics and Nutrition, AHC 1 450, 11200 S. W. 8th Street, Miami, FL 33199, USA; 2 Florida International University, Robert Stempel College of Public Health and Social Work, Department of Dietetics and Nutrition, AHC 1 435, 11200 S. W. 8th Street, Miami, FL 33199, USA

## Abstract

**Background:**

Diabetes is a global epidemic. Cardiovascular disease (CVD) is one of the most prevalent consequences of diabetes. Nutrition is considered a modifiable risk factor for CVD, particularly for individuals with diabetes; albeit, there is little consensus on the role of carbohydrates, proteins and fats for arterial health for persons with or without diabetes. In this study, we examined the association of macronutrients with arterial pulse pressure (APP), a surrogate measure of arterial health by diabetes status and race.

**Methods:**

Participants were 892 Mexican Americans (MA), 1059 Black, non-Hispanics (BNH) and 2473 White, non-Hispanics (WNH) with and without diabetes of a weighted sample from the National Nutrition and Health Examination Survey (NHANES) 2007-2008. The cross-sectional analysis was performed with IBM-SPSS version 18 with the complex sample analysis module. The two-year sample weight for the sub-sample with laboratory values was applied to reduce bias and approximate a nationally, representative sample. Arterial stiffness was assessed by arterial pulse pressure (APP).

**Results:**

APP was higher for MA [B = 0.063 (95% CI 0.015 to 0.111), p = 0.013] and BNH [B = 0.044 (95% CI 0.006 to 0.082), p = 0.018] than WNH, controlling for diabetes, age, gender, body mass index (BMI), fiber intake, energy intake (Kcal) and smoking. A two-way interaction of diabetes by carbohydrate intake (grams) was inversely associated with APP [B = -1.18 (95% CI -0.178 to -0.058), p = 0.001], controlling for race, age, gender, BMI, Kcal and smoking. BNH with diabetes who consumed more mono-unsaturated fatty acids (MUFA) than WNH with diabetes had lower APP [B = -0.112 (95%CI-0.179 to -0.045), p = 0.003] adjusting for saturated fatty acids, Kcal, age, gender, BMI and smoking.

**Conclusion:**

Higher MUFA and carbohydrate intake for persons with diabetes reflecting lower APP may be due to replacement of saturated fats with CHO and MUFA. The associations of APP with diabetes, race and dietary intake need to be confirmed with intervention and prospective studies. Confirmation of these results would suggest that dietary interventions for minorities with diabetes may improve arterial health.

## Background

The prevalence of diabetes and its health consequences has been growing disproportionately in the U.S. and throughout the world. Cardiovascular disease (CVD) is the most prevalent and detrimental complication of diabetes accounting for about 65% of deaths from heart disease and stroke [1-3]. Death rates among adults with diabetes were 2-4 times higher from heart disease and about 2.8 times greater from stroke, when compared to individuals without diabetes [4,5].

Arterial stiffening which leads to CVD is consistently observed across all age groups for individuals with diabetes [6]. A complex series of structural, cellular and hormonal changes contribute to the lack of resilience of the vascular wall, promoting arterial stiffening [6]. A possible link between diabetes and CVD could be due to the pathological changes in the endothelium, at the microvascular level that leads to the stiffening of arteries in persons with diabetes. This stiffening reduces the artery's ability to expand and recoil leading to a progressive rise in systolic blood pressure (SBP) and decrease in diastolic blood pressure (DBP) [7,8]. The difference between SBP and DBP is referred to as arterial pulse pressure (APP). A high APP is indicative of increased arterial stiffness that promotes further stiffening of the arteries, and has been associated with CVD morbidity and mortality [6,9]. Arterial pulse pressure is easily calculated by the difference between systolic and diastolic blood pressure and is a non-invasive, convenient, surrogate measure of arterial stiffness [10].

For epidemiological studies and clinical trials invasive methods or the use of expensive instruments (such as those measuring pulse wave velocity) may not be feasible; yet, measures of arterial stiffness for participants by disease state and social characteristics serve public health interests. Particularly since CVD is a leading cause of death and cardiovascular risk factors such as age, obesity, diabetes, hypertension, hypercholesterolemia and smoking have been associated with increased arterial stiffness and systemic endothelial dysfunction [11]. Arterial pulse pressure has been correlated to endothelial function in hypertensive patients [12] and with pulse wave velocity and endothelial function in healthy participants [11]. Therefore, APP may be a feasible method for screening endothelial function and arterial health for epidemiological studies.

While it has been established that diet and dietary pattern play a role in the prevention of hypertension, the role of diet on APP has not been determined. The proportion and type of macronutrients (carbohydrate (CHO), protein and fat) may be associated with arterial health. Current dietary guidelines regarding the type and proportion of macronutrient for persons with diabetes by the American Diabetes Association (ADA) have not been consistently supported by the literature. While ADA advocates a high fiber, high CHO diet, studies have found beneficial diabetes outcomes with high protein, low CHO diets. Several clinical trials have reported dietary composition, mainly proportion of calories from CHO, have an effect on blood pressure for normo- and hypertensive adults [13-16]; however, few studies compare these effects for persons with type 2 diabetes. The highest dietary type and source of monounsaturated fatty acid (MUFA), is from oleic acid found primarily in unrefined (virgin) olive oil, followed by high oleic-sunflower and safflower oil and canola oil [17]. A random-effect model of ten studies found diets high in CHO and low in MUFA were associated with a higher SBP {2.6 (0.4, 4.7) mm Hg; p = 0.002} and DBP {1.8 (0.01, 3.6) mm Hg; p = 0.05} [18]. However, a review of epidemiological studies assessing the effect of MUFA for normal populations on blood pressure revealed conflicting results [17]. Even though there were few epidemiological studies from Mediterranean countries, the results all indicated that MUFA intake was inversely associated with blood pressure [17]. Patients with type 2 diabetes (N = 15) from a 3-week, crossover diet intervention were found to have reduced blood pressure with a 30% MUFA/30% CHO diet as compared to a 10%MUFA/50% CHO diet [19]. Current analysis in our laboratory of Hispanics by diabetes status reported an inverse relationship between carbohydrate intake and APP for individuals with diabetes; while protein intake was inversely associated with APP for persons without diabetes. Based on these finding, our objective was to assess APP and its association with macronutrient by diabetes status for a multiethnic sample. We hypothesized that CHO, with respect to total calories and fiber, would be positively associated with higher APP in individuals with diabetes regardless of gender and ethnicity. We further hypothesized that MUFA would be inversely associated with APP for individuals regardless of diabetes status, gender and ethnicity.

## Methods

This study was a cross-sectional, secondary analysis of National Health and Nutrition Examination Survey, NHANES 2007-2008 [20] adults' ≥ 21 with and without diabetes from three ethnicities: Mexican American (MA); Black, non-Hispanic (BNH); and White, non-Hispanic (WNH). NHANES has 5 categories for ethnicity, the sample size for individuals with diabetes in 2 categories: "Other Hispanic" and "Other" was insufficient for multiple comparisons and were not used in the analysis. Participants were (N = 4766) adults with (n = 622) and without diabetes (n = 4144) and for whom dietary and anthropometric data were available. The two-year sample weight for laboratory analyses was used to achieve a non-biased, representative sample of the United States population.

### Data collection

Raw data were extracted from datasets collected from the NHANES 2007-2008 [20] available for public use. Data were available for use without additional informed consent, since informed consent was obtained by NHANES and this was a secondary analysis of unrestricted data. This survey contains data for 10,149 individuals of all ages. The NHANES survey design is a stratified, multistage probability sample of the civilian non-institutionalized U.S. population. The stages of sample selection are as follows: 1) Primary Sampling Units (PSUs), which are counties or small groups of contiguous counties; 2) segments within PSUs (a block or group of blocks containing a cluster of households); 3) households within segments; and 4) one or more participants within households. A total of 15 PSUs were visited during a 12-month period.

For the household interview, participants were those who understood, agreed to and signed an interview consent for the household interview portion of the survey. After the household interview was completed, all interviewed persons were asked to complete the health examination component. Those who agreed to participate were asked to sign additional consent forms for the NHANES health examination component. Informed consent was obtained from each subject in the study. The health examinations were conducted in mobile examination centers (MECs); the MECs provide a standardized environment for the collection of high quality data.

The nutrition assessment was conducted in a private room in the MEC, by trained dietary interviewers fluent in Spanish and English. A standard set of measuring guides were used to help participants estimate food portions that they consumed. The first day face-to face interview was used rather than the second day 24-hour recall since the second day interview was conducted by telephone and values were not available for all participants. In addition, the telephone interview process may introduce subject bias since the conditions were not the same for all subjects telephoned. Finally, comparing subjects telephoned to those interviewed introduces an extraneous variable when combining both 24-hour recalls for analysis.

#### Diabetes status variable

Participants who answered "yes" to the question 'have you ever been told by a doctor or health professional that you have diabetes or sugar diabetes' were classified as having diabetes and those who answered "no" were classified as not having diabetes.

#### Ethnicity and adult status

Filters were applied to select individuals of age 21 years or more and belonging to either MA, BNH or WNH ethnicities.

#### Demographic and anthropometric variables

There were insufficient numbers of persons at the highest education level by ethnicity (based on the estimated marginal means) so the education variable was collapsed from 5 to 4 categories. An obesity variable was constructed for body mass index (BMI) where BMI ≥ 30 kg/m^2 ^and was transformed by natural logarithm to achieve linearity.

### Dietary variables

The first day 24 hour recall were used to construct all dietary variables. The first day 24 hour recall was chosen since it was administered in person; whereas the second day 24 hour recall was conducted by telephone. Monounsaturated fatty acid (MUFA) and saturated fatty acid (SFA) intakes were transformed by the square root to achieve linearity. Carbohydrate and protein were transformed by natural-logarithm.

### Major outcome variable

The dependent variable, APP was calculated by subtracting the first SBP and DBP readings for all participants. The first reading was chosen rather than subsequent reading since values for all participants were available. Since a true difference between systolic and diastolic pressure was sought, actual readings were subtracted rather than the average.

### Statistical analysis

Descriptive statistics and frequencies were run by complex sample analysis for participant characteristics. Separate regression models for dietary analysis: fats, protein, carbohydrate by diabetes status, ethnicity and gender were performed for APP by complex sample analysis (weighted samples) using the General Linear Model. Continuous variables were tested by Q-Q plots and transformed if necessary to achieve linearity. Known confounders such as age, gender, smoking, BMI and Kcal were used for adjustment in all models. Possible confounders such as poly-unsaturated fatty acids (PUFA), blood pressure medication, physical activity, dietary sodium and dietary potassium were included in the full model and eliminated in the final model since they were not significant. Statistical analyses were conducted with IBM-SPSS version 18 by the complex sample module. It has been suggested by the National Center for Health Statistics [21] to select the sample weight with the smallest sub-sample based on the major variables. The sample weight from the mobile examination center (MEC) for laboratory was used since it contained the smallest sample size than demographics and dietary sub-samples. Since the dietary sub-sample was close in size to the laboratory sub-sample, analysis were also conducted with the dietary sample weight for comparison. The direction of all relationships was the same and magnitudes were not significantly different with either method. Sample weights were used to achieve an unbiased, nationally representative sample of non-institutionalized citizens of the U.S. The group WNH was use as a reference for race so that BNH and MA were compared to WNH. For all analyses, p-value of < 0.05 was considered significant.

## Results

Descriptive characteristics of the weighted sample are presented by race in Table 1. Statistical analyses by the complex GLM, analysis of covariance (ANCOVA) for the weighted proportion, were conducted to determine significant differences for each continuous variable. The following variables were significantly different by race: APP, age, diabetes and smoking status, intakes of dietary fiber and saturated fat. Carbohydrate and MUFA intakes did not vary by race. The MA group consisted of a higher percentage of males; while, BNH and WNH were composed of a higher percentage of females. Black, non-Hispanics had the highest percentage of reported cases of diabetes.

**Table 1:** Characteristics of the participants*

Comparison^†^	Mexican Americans N = 892	Black non-Hispanic N = 1059	White non-Hispanic N = 2473	P MA/ WNH	P BNH/ WNH
**Continuous variables**	**Mean (lower-upper 95% CI) SE**				

Arterial pulse pressure	51.0 (48.2-53.9) 1.34	53.4 (51.6-55.3) 0.86	52.4 (51.1-53.7) 0.62	0.329	0.285
Carbohydrate (gm)	268.3 (258.6-277.9) 4.54	250.4 (240.0-260.8) 4.91	260.4 (253.2-267.7) 3.43	0.203	0.074
Fiber (gm)	18.2 (17.2-19.2) 0.45	13.2 (12.6-13.9) 0.31	16.1 (14.7-17.4) 0.63	0.006	0.001
Monounsaturated fat (gm)	30.1 (28.1-32.1) 0.94	30.8 (29.3-32.4) 0.72	31.2 (30.0-31.2) 0.56	0.342	0.684
Saturated fat (gm)	25.4 (23.7-27.1) 0.81	25.8 (24.1-27.5) 0.79	28.3 (27.3-29.3) 0.46	0.006	0.017
Total daily calories	2192 (2101-2283) 43.0	2108 (2015-2201) 43.8	2186 (2128-2244) 27.5	0.902	0.151
Age (years)	40.2 (38.5-42.0) 0.83	44.5 (43.7-46.0) 0.54	48.9 (47.9-50.0) 0.49	< 0.001	< 0.001
BMI (kg/m^2^)	29.5 (28.8-30.2) 0.32	30.2 (29.6-30.8) 0.29	28.4 (27.9-28.9) 0.25	0.023	< 0.001

**Categorical variables**	**N (%) SE^‡^**			**χ^2^**	**P**

Diabetes (Yes)	121 (7.9) 0.8	213 (14.2) 1.2	288 (8.0) 1.1	40.8	< 0.001
No	838 (92.1)	953 (85.8)	2353 (92.0)	-	-
Current Smoker (Yes)	155 (17.7) 1.9	300 (26.6) 2.1	640 (23.3) 1.7	97.9	< 0.001
No	803 (82.3)	865 (73.4)	2002(76.7)	-	-
Gender				2.60	0.272
• Male	459 (53.9) 2.4	557 (44.4) 1.5	1342 (47.8) 0.7	-	-
• Female	501 (46.1)	609 (55.6)	1301 (52.2)	-	-

*Abbreviations: *BNH = Black, non-Hispanic, MA = Mexican American, WNH = White, non-Hispanic; BMI = body mass index.

*Laboratory sample weights were used in all analyses. Actual sample numbers are presented with their weighted results expressed as standard error range in the 95% confidence intervals.

**^† ^**MA and BNH were compared to WNH for all analyses. Variables with p-values for each group (MA and BNH) that are < 0.05 are significantly different than WNH.

Race and diabetes status, were independent predictors of APP, adjusting for age, gender, BMI, Kcal and smoking (Table 2). Having diabetes and being BNH was associated with a lower APP than for WNH with diabetes. Next a series of models for protein, carbohydrate and fat intakes were conducted with APP as the dependent variable. The final macronutrient models indicated that the dietary variables of CHO and MUFA interacted with diabetes status in determining APP. Protein was not a significant predictor of APP; however, CHO and MUFA were associated with APP. The reduced models for APP regressed on CHO (Model 1) and MUFA (Model 2) are shown in Table 3. For Model 1, the simple-sugars-group was not significantly different by diabetes status or race and was eliminated for the reduced model. Controlling for CHO, fiber, age, gender, smoking, BMI and Kcal, race by itself no longer predicted APP in the CHO or in the MUFA models. Instead, the 2-way interaction of diabetes with race was significant for APP (Model 1, p = 0.006; Model 2, p = 0.007). Specifically, BNH with diabetes had lower APP than WNH with diabetes (Models 1 and 2, p = 0.003); whereas MA with diabetes did not have significantly different APP compared to WNH (Model 1, p = 0.171, Model 2, p = 0.088). The effect of CHO and MUFA were modified by diabetes status for determining an association with APP and was independent of race. Having diabetes and consuming lower CHO was associated with higher APP, adjusting for fiber, calories, race, BMI, smoking, gender, and age (p = 0.001). Similarly, having diabetes and consuming lower MUFA was associated with higher APP (p = 0.011).

**Table 2:** Diabetes status and race as factors of APP*

Independent Variables	B-unstandardized coefficient (95% CI)	SE	P*
Diabetes (Yes)	0.113 (0.075, 0.151)	0.018	< 0.001

Race	-	-	< 0.001
MA	0.057 (0.11, 0.103)	0.022	0.019
BNH	0.062(0.021, 0.104)	0.020	0.006
WNH (reference) **^† ^**			
Diabetes (yes)*RACE [F(2,15) = 4.54]	-	-	0.029
Diabetes (yes) MA	-0.45(-0.122, 0.032)	0.036	0.232
Diabetes (yes) BNH	-0.096(-0.168,-0.023)	0.034	0.013
WNH (reference) **^‡^**			
*Covariates*			
Current Smoker (yes)	0.026 (0.006, 0.046)	0.009	0.015
Age	0.008 (0.008, 0.009)	0.0001	< 0.001
Gender	0.015 (-0.015,0.045)	0.014	0.312
BMI (m/kg^2^)	0.048(-0.022, 0.118)	0.033	0.163
Kcal	-0.001(-0.002, 0.001)	0.001	0.214

Dependent Variable: APP Model summary: R^2 ^= 0.178; F (10,7) = 52.7; p < 0.001			

*Abbreviations: *MA = Mexican American; BNH = Black, non-Hispanic;

WNH = White, non-Hispanic; APP = arterial pulse pressure; Kcal = kilocalories per day (energy intake); BMI = body mass index.

* Complete data were available for N = 4237 participants and sample weights were applied. BMI was natural log-transformed and Kcal was transformed by the square root.

**^† ^**MA and BNH are compared to WNH regarding APP and have higher APP than WNH (positive coefficient).

**^‡ ^**MA and BNH with diabetes are compared to WNH with diabetes and have lower APP (negative coefficients, significant for MA, only)

**Table 3:** Effect of Diabetes status, race and diet on APP*

Model 1 Carbohydrate/Fiber	B-unstandardized coefficient (95% CI)	SE	P
Diabetes (yes)	0.744 (0.436,1.05)	0.145	< 0.001
RACE [F(2,15) = 0.539]	-	-	0.594
MA	0.060 (0.015, 0.106)	0.021	0.012
BNH	0.059 (0.019, 0.099)	0.019	0.006
WNH (reference) **^†^**			
Diabetes*RACE [F(2,15) = 7.39]	-	-	0.006
Diabetes (yes) MA	-0.054(-0.134, 0.026)	0.038	0.171
Diabetes(yes) BNH	-0.112(-0.179, -0.045	0.032	0.003
Diabetes(yes) WNH (reference) **^‡^**	-	-	-
CHO (gm)	0.051 (-0.005, 0.106)	0.026	0.071
Fiber (gm)	-0.014 (-0.029, -0.0003)	0.007	0.050
Kcal	-0.002(-0.004, 0.001)	0.001	0.161
Diabetes (yes) *CHO^‡‡^	-0.118 (-0.178, -0.058)	0.028	0.001
Current Smoker (yes)	0.022 (0.001, 0.043)	0.010	0.041
Age (yrs)	0.008 (0.007,0.009)	0.0001	< 0.001
Gender (male)	0.022 (-0.014, 0.058)	0.017	0.208
BMI (m/kg^2^)	0.045(-0.024,0.114)	0.032	0.182

Model summary: R^2 ^= 0.184; F(13,4) = 36.5, p < 0.001			

**Model 2 MUFA/SFA**	**B (95% CI)**	**SE**	**P**
Diabetes (Yes)	0.299 (0.195, 0.403)	0.049	< 0.001
RACE [F(2,15) = 0.351]			0.710
MA	0.058 (0.012, 0.104)	0.022	0.017
BNH	0.065 (0.022, 0.107)	0.020	0.005
WNH (reference)			
Diabetes*RACE [F(2,15) = 7.12]	-	-	0.007
Diabetes (yes) MA	-0.063(-0.137, 0.001)	0.035	0.088
Diabetes (yes) BNH	-0.112(-0.179,-0.044)	0.032	0.003
Diabetes (yes) WNH (reference) **^‡ ^**			
MUFA	-0.019(- 0.040,0.002)	0.010	0.072
SFA	0.010(-0.006,0.026)	0.008	0.186
Kcal	0.001(-0.002, 0.003)	0.001	0.547
Diabetes (yes) *MUFA^‡‡^	-0.035 (-0.055, -0.014)	0.010	0.011
Current Smoker (Yes)	0.024 (0.004, 0.045)	0.010	0.020
Age (yrs)	0.008 (0.007, 0.009)	0.0001	< 0.001
Gender (male)	0.021(-0.013, 0.056)	0.016	0.208
BMI (m/kg^2^)	0.054(-0.18,0.126)	0.034	0.134
Model summary: R^2 ^= 0.184; F(13, 4) = 72.9, p < 0.001			

*Abbreviations: *APP = arterial pulse pressure; MA = Mexican Americans; BNH = Black, non-Hispanics; WHN = White, non-Hispanics; MUFA = monounsaturated fatty acids; SFA = saturated fatty acids; Kcal = kilocalories per day (energy intake); BMI = body mass index.

* Complete data were available for N = 4236 participants and sample weights were applied. BMI was natural log-transformed and Kcal was transformed by the square root.

**^† ^**MA and BNH are compared to WNH regarding APP and have higher APP than WNH (positive coefficient).

**^‡ ^**MA and BNH with diabetes are compared to WNH with diabetes and have lower APP (negative coefficients, significant for MA, only)

**^‡‡ ^**All participants with diabetes that consume higher CHO and higher MUFA have lower APP than those without diabetes.

## Discussion

This study examined a non-invasive, surrogate indicator of arterial health, APP and its relationship to diet for individuals with and without diabetes. To address the gap in the literature concerning diet and arterial health, APP was assessed with respect to diet for a nationally representative sample of persons with and without diabetes. Although several studies and a meta-analysis indicate higher protein, lower CHO diets have a more favorable effect on blood pressure, there are contradictory findings regarding the proportion of dietary carbohydrate with respect to daily caloric consumption for persons with diabetes. There are limited studies of MUFA and blood pressure and no known studies of MUFA and APP.

Our study found differences in APP by diabetes status with MUFA and CHO. Specifically, lower intake of CHO and MUFA were associated with higher APP for persons with diabetes as compared to persons without diabetes, independent of race. These associations may be due, in part, to the replacement of SFA and/or trans-fatty acids (TFA) with CHO and/or MUFA. The effect of SFA on CVD has been well-established in the literature. In the last decade, TFA have been implicated as a CVD risk factor. Although rare in nature, TFA, ubiquitous in margarines, baked goods, shortenings, fried and packaged foods, are partially hydrogenated vegetable oils where approximately 30-60% of the naturally occurring cis unsaturated double bonds are converted into trans-unsaturated double bonds [22]. TFA adversely affecting lipid profile in several randomized clinical trials and may promote endothelial dysfunction [[22] and references therein]. Our findings that PUFA was not associated with MUFA and APP were in accordance with a 14 year follow-up study of coronary heart disease (CHD) in men (N = 45,722) initially free of known CVD [23]. The authors found MUFA intake and CHD were independent of PUFA intake [23].

Diets high in CHO and low in MUFA were associated with a higher SBP for a random-effect model of ten studies [18]. This meta-analysis combined one pre-hypertensive, one hyperlipidemic, four normotensive and four type 2 diabetes studies. Although the investigators used the random-effect model which controlled for disease state, they did not compare individuals with to those without type 2 diabetes. Rasmussen et al [19] reported a reduction in SBP and DBP with no change in lipids for a high fat, a high MUFA diet as compared to an isocaloric and high CHO diet for individuals with type 2 diabetes. High protein/low CHO composition diets were associated with improved glycemic index, blood pressure and weight loss in persons with diabetes in several studies [13-16]. Yet, high protein/low CHO diets may not prove to be beneficial for arterial health for individuals with certain metabolic diseases (such as type 2 diabetes or metabolic syndrome) since the long-term effect of these diets are not known [24]. Glycemic improvement was most likely a result of weight loss rather than the particular diet. Isocaloric diets that promote weight maintenance as opposed to weight loss may be more indicative of macronutrient consumption, blood pressure and arterial health.

While there are limited studies with respect to arterial health and carbohydrate intake, numerous studies have been conducted comparing fatty acid type with endothelial functioning. However, few studies of fat-type have been conducted in individuals with diabetes. It has been shown that endothelial function is affected by fatty acid content in blood [25] and ratio of polyunsaturated to saturated (P/S) fatty acids [26]. The mechanisms involving MUFA on blood pressure and arterial health are less clear. There is evidence that endothelial products responsible for thrombogenesis were diminished by a diet high in MUFA with (22%) MUFA/6% PUFA/10% SFA as compared to a low fat diet composed of 12% MUFA/6% PUFA/10% SFA for healthy males [27]. Endothelial function, assessed by flow-mediated dilation, for men and women with diabetes was not significantly altered by a MUFA-rich meal; but, was impaired by a meal high in SFA [28]. These results suggest that MUFA, in the form of olive oil, may have a protective effect on endothelial function in individuals with type 2 diabetes [28]. Olive oil, in addition to oleic acid, contains many polyphenols in different amounts and types from other MUFA oils and these polyphenols may play a role in enhancing endothelial function by promoting the formation of nitric oxide and suppressed the formation of adhesion molecules [23-29]. One reason why there are conflicting results concerning MUFA intake and blood pressure may be attributed to the type of MUFA [17]. Oleic acid may be responsible for the mechanism promoting blood pressure regulation [17] as well as endothelial function.

A limitation of this study was its cross-sectional nature; and therefore, causality cannot be inferred. Diabetes diagnosis was by self-report and not by medical records; albeit, the American Diabetes Association[30] and the World Health Organization [31] endorses the determination of diabetes hyperglycemia and/or self-reported diabetes. Although the results were from data weighted to a nationally representative sample, this comparison was for three major ethnic groups and did not include Asians and Hispanics other than Mexican Americans. Mexican Americans constitute a larger proportion of non-U.S. citizens and undiagnosed cased of diabetes than BNH and WHN; therefore, the weighted sample might not have take into account these differences. We were not able to assess TFA intake and it may have been a confounder with CHO and APP. Finally, the sources of MUFA were not quantified in this study and variations in antioxidant properties may have been a confounder in the association of MUFA with APP.

## Conclusion

This is the first study that demonstrated relationships among CHO and MUFA intakes by diabetes status and race with APP. Arterial stiffness is a major surrogate indicator of CVD that is associated with complications of diabetes. This study suggests the balance between macronutrients for arterial health may differ by diabetes status and race. Both MA and BNH had higher APP than WNH, controlling for current smoking and age. Clinical trials and prospective studies are needed to determine if dietary CHO and MUFA intakes may be beneficial for APP for persons with diabetes or if the replacement of SFA and TF with CHO and MUFA is protective of arterial health. The inverse relationship of MUFA intake and APP was observed for Blacks with diabetes, a population who are disproportionately affected by elevated BP and diabetes complications. Identifying the role of macronutrients in arterial health for individuals with diabetes, would address an important public health issue since compromised arterial health may be prevented by dietary modifications of macronutrient balance.

## Abbreviations

APP: arterial pulse pressure; BMI: body mass index; BNH: Black, non-Hispanic; CDC: Center for Disease Prevention and Control; CHD: coronary heart disease; CHO: carbohydrate; CI: confidence interval; CVD: cardiovascular disease; DBP: diastolic blood pressure; DHNES: Division of Health and Nutrition Examination Surveys; MA: Mexican American; MEC: mobile examination center; MUFA: monounsaturated fatty acids; NCHS: National Center for Health Statistics; NHANES: National Health and Nutrition Examination Survey; P/S: polyunsaturated to saturated; PSU: Primary Sampling Units; PUFA: polyunsaturated fatty acids; SBP: systolic blood pressure; SFA: saturated fatty acid; WNH: White, non-Hispanic.

## Competing interests

The authors declare that they have no competing interests.

## Authors' contributions

JAV and FGH contributed to the design of the study, manuscript development, interpretation of the data and editing. JAV participated in the data analysis. Both authors have read and approved the final manuscript.
